# Connecting Climate Change Mitigation to Global Land Regeneration, Doubling Worldwide Livestock, and Reduction of Early Deaths from Noncommunicable Diseases

**DOI:** 10.7759/cureus.33253

**Published:** 2023-01-02

**Authors:** David K Cundiff

**Affiliations:** 1 Internal Medicine and Global Health, Independent Medical Researcher, California, USA

**Keywords:** greenhouse gases, regenerative/organic agriculture, global livestock, climate change, noncommunicable disease

## Abstract

Aim and background

This article aims to link early deaths due to diet-related noncommunicable diseases at the global level, low animal food intake, primarily in developing countries, regenerative/organic agriculture, worldwide food security, and global warming mitigation. On statistically modeling Global Burden of Disease (GBD) risk factor and health outcome data, the unexpected finding was that early deaths (death before age 70) per year per 100k population due to noncommunicable diseases (NCDs, such as coronary artery disease, emphysema, liver failure, kidney failure, and cancers) were much higher in cohorts with low consumption of animal-sourced foods (processed meat, red meat, dairy, fish, poultry, eggs, and saturated fats). Relatively low NCD rates are associated with high animal food consumption. This unexpected finding led to exploring the implications of climate change.

Methods

I critiqued the Intergovernmental Panel on Climate Change’s (IPCC’s) definitions of “sustainability in land management, sustainable intensification (of agriculture), climate-smart agriculture,” and “sustainability-focused socioeconomic pathway 1 (SSP1)”-the most climate-favorable scenario that the IPCC modeled. I modeled doubling the global livestock together with global regenerative/organic agriculture compared with the IPCC’s SSP1, using the IPCC’s mean 2010-2019 global anthropogenic greenhouse gas emissions (GHGs) as the baseline for comparison.

Results

This study found that all the IPCC’s agricultural land-related definitions of interest were aspirational without detailing the farming methods used and those not allowed. The IPCC’s land management-related definitions differed from the same or similar terms in the literature. The status quo net global agriculture and other land use GHGs (2010-2019) totaled 11.9 ± 4.4 gigatonnes (GT) carbon dioxide equivalent per year (11.9 ± 4.4 GTCO_2_-eq yr^-1^). The IPCC’s modeling of the SSP1 scenario reduced GHGs to 3 GTCO_2_-eq yr^-1^ by 2050. Transitioning to global regenerative/organic agriculture (5 billion hectares) and doubling the global livestock for human consumption and agricultural land fertilization corresponded to net global GHGs = -24.1 GTCO_2_-eq yr^-1^ for 2-3 decades, totaling -482 to -723 GTCO_2_-eq of CO_2_ sequestration.

Conclusions

Doubling global livestock combined with worldwide regenerative/organic agriculture has the potential to mitigate climate change significantly more than SSP1 while providing global food security by reversing land degradation. Worldwide transitioning from intensive industrial agriculture that degrades land to regenerative/organic agriculture that sequesters CO_2_ in soil ​​​​​and doubling global livestock would require initial support with finances, resources, and additional workers for farms in both developing and developed countries. Subsequently, farms and farmers would be sustainably self-supporting with food sales. Retaining the existing farm workers and attracting hundreds of millions more workers would likely require transitioning most agricultural lands into worker-owned cooperatives.

## Introduction

As volunteer collaborators with the Institute of Health Metrics and Evaluation (IHME), Chunyi Wu and I formatted, explored, and modeled the IHME Global Burden of Disease (GBD) risk factor and health outcome data from a population-weighted GBD database of 7846 cohorts, representing about 7.8 billion people in 195 countries [1]. This analysis was done in conjunction with the overall GBD study [2].

Unexpectedly, we found that the lower the amount of animal-sourced foods (processed meat, red meat, dairy, fish, poultry, eggs, and saturated fats), the higher the rate of early deaths (death before age 70) per year/100k population from over 100 noncommunicable diseases (NCDs, e.g., coronary artery disease, emphysema, liver cirrhosis, cancers, etc.). NCDs worldwide negatively correlated with animal food consumption (mean NCDs = 1428 deaths/year/100k, mean animal food intake = 264.68 kilocalories/day (kcal/day), r= -0.287 95% CI: -0.307 to -0.266, P<0.0001). However, in wealthy countries with high animal food consumption, NCD early death is positively correlated with animal food intake (e.g., in the United States: mean NCDs = 1197 deaths/year/100k, mean animal food intake= 700.66 kcal/day and mean animal food intake positively correlated with NCDs: r= 0.731 95% CI 0.677 to 0.777, P < 0.0001 [1].

From this analysis, our medical journal preprint concluded, “GBD data suggest that at least doubling the average worldwide animal food production and consumption (i.e., mean animal food 581.4 kcal/day versus mean worldwide=242 kcal/day) and distributing it more evenly predicts for a dramatic reduction of worldwide NCD” [1].

This surprising finding that low animal foods intake is associated with higher early deaths from NCDs led me to consider the implications of these findings on the Intergovernmental Panel on Climate Change (IPCC) report related in part to agricultural methods of sequestering carbon in the ground [3]. 

## Materials and methods

This paper critiques the IPCC’s definitions of terms related to agriculture: “sustainable land management,” sustainable intensification (of agriculture), and climate-smart agriculture. The IPCC authors modeled five scenarios for remediating agricultural land, forests, and other lands. “Sustainability-focused shared socioeconomic pathway 1 (SSP1) is the IPCC’s scenario most favorable to the environment and would produce the lowest estimated amount of greenhouse gas emissions (GHGs) [4]. This paper reports the IPCC's definition of SSP1 and critiques that definition. It also reports the IPCC's determination of the expected global agricultural, forest and other land uses GHGs that would be associated with SSPI. Finally, this presents an IPCC report referenced study that appears to be inconsistent with the mitigation of climate change. 

In contrast with the IPCC modeling of SSP1, this article presents the estimated agricultural GHGs associated with transitioning to doubling the global livestock and transitioning to global regenerative/organic agriculture. This paper contrast the modeling of net GHGs attributable to doubling the global livestock and transitioning to global regenerative/organic agriculture to the IPCC's modeling of the status quo GHGs from 2010-2019.

According to the IPCC, "Net GHG emissions in this report refer to release of greenhouse gases from anthropogenic sources minus removals by anthropogenic sinks, for those species of gases that are reported under the common reporting format of the United Nations Framework Convention on Climate Change (UNFCCC): CO_2_ from fossil fuel combustion and industrial processes (CO_2_-FFI); net CO_2_ emissions from land use, land-use change and forestry (CO_2_-LULUCF); methane (CH_4_); nitrous oxide (N_2_O); and fluorinated gases (F-gases) comprising hydrofluorocarbons (HFCs), perfluorocarbons (PFCs), sulphur hexafluoride (SF_6_), as well as nitrogen trifluoride (NF_3_)" [3]. This analysis includes the major GHGs: C0_2_, CH_4_, and NO_2_, but does not include the relatively minor gases.

Regarding the analyses of the GHGs, the IPCC report said, "GHG emission metrics are used to express emissions of different greenhouse gases in a common unit. Aggregated GHG emissions in this report are stated in CO_2_-equivalent (CO_2_-eq) using the Global Warming Potential with a time horizon of 100 years (GWP100) with values based on the contribution of Working Group I to the AR6. The choice of metric depends on the purpose of the analysis, and all GHG emission metrics have limitations and uncertainties, given that they simplify the complexity of the physical climate system and its response to past and future GHG emissions" [3].

With the IPCC's modeling of GHGs due to agriculture, forests and other land uses, the global total gigatonnes of net anthropogenic gigatonnes of carbon dioxide equivalent (GTCO_2_-eq) approximately equals the anthropogenic GTCO_2_ + equivalence of GTCH_4 _+ equivalence of GTNO_2_. The overall net gigatonnes of GHGs also accounts for the gigatonnes of natural carbon sink which absorbs CO_2_.

With the above methodology, this article also derives the total estimated global GHGs associated with doubling animal-sourced foods, transitioning to global regenerative/organic agriculture, optimally managing forests, and eliminating the burning of fossil fuels worldwide.

## Results

Critiques of the IPCC’s definitions of terms related to agriculture

The IPCC report defined sustainable land management as “The stewardship and use of land resources, including soils, water, animals and plants, to meet changing human needs, while simultaneously ensuring the long-term productive potential of these resources and the maintenance of their environmental functions” [5,6]. This definition does not include what agricultural methods are or are not used in sustainable land management. The United Nations (UN) determined that “75% of the terrestrial environment is ‘severely altered’ to date by human actions (marine environments 66%) and there is a 47% reduction in global indicators of ecosystem extent and condition against their estimated natural baselines, with many continuing to decline by at least 4% per decade” [7]. The IPCC itself reported, “Lack of action to address land degradation will increase emissions and reduce carbon sinks and is inconsistent with the emissions reduction required to limit global warming to 1.5°C or 2°C. (high confidence)” [8]. Sustaining the current soil status of soil organic matter, overall fertility, and ecosystem services is therefore not adequate for humanity even if global indicators of ecosystem extent and condition do not deteriorate further beyond their already highly degraded states.

The IPCC report defined sustainable intensification (of agriculture) as “Increasing yields from the same area of land while decreasing negative environmental impacts of agricultural production and increasing the provision of environmental services” [9]. Nowhere in the definition of sustainable intensification (of agriculture) did it say what methodologies are or are not included in agricultural intensification. According to the Encyclopedia Britannica [10], intensive agriculture involves chemical fertilizers, insecticides, fungicides, herbicides, monocropping, and confined animal feeding operations. According to Wikipedia, “Intensive agriculture, also known as intensive farming (as opposed to extensive farming), conventional, or industrial agriculture, is a type of agriculture, both of crop plants and of animals, with higher levels of input and output per unit of agricultural land area. It also involves increased use of fertilizers, plant growth regulators, pesticides, antibiotics for livestock” [11]. The United States Department of Agriculture defined the sustainable intensification of agriculture without detailing the methodologies as follows, “Sustainable crop production intensification provides opportunities for optimizing crop production per unit area, taking into consideration the range of sustainability aspects including potential and/or real social, political, economic and environmental impacts” [12]. The Factory Farming Awareness Coalition gave a negative characterization of intensive agriculture, “Intensive agriculture is a method of farming that uses large amounts of labor and investment to increase the yield of the land. In an industrialized society, this typically means the use of pesticides, fertilizers, and other chemicals that boost yield, and the acquisition and use of machinery to aid planting, chemical application, and picking. In theory, this reduces the amount of land needed for an economically viable farm to grow crops or raise animals. However, in countries such as the United States and Canada, these methods are often used to overproduce products as companies attempt to increase their market share. Profit is then diminished so that farmers must continue overproducing in order to stay economically viable, and often seek compensation for low profits via government subsidies” [13]. 

The IPCC report defined climate-smart agriculture, “Climate-smart agriculture (CSA) is an approach that helps to guide actions needed to transform and reorient agricultural systems to effectively support the development and ensure food security in a changing climate. CSA aims to tackle three main objectives: sustainably increasing agricultural productivity and incomes, adapting and building resilience to climate change, and reducing and/or removing greenhouse gas emissions, where possible (Food and Agriculture Organization of the United Nations {FAO}, 2018)” [14]. In this definition, the phrase, “reducing and/or removing greenhouse gas emissions, where possible,” indicates that “increasing agricultural productivity and incomes” take precedence. Again, agricultural methods that are and are not used in climate-smart agriculture were not detailed.

The IPCC report defined sustainability-focused shared socioeconomic pathway 1 (SSP1), “Sustainability in land management, agricultural intensification, production and consumption patterns result in reduced need for agricultural land, despite increases in per capita food consumption. This land can instead be used for reforestation, afforestation, and bioenergy” [4]. Again, the methods permitted and not permitted in SSP1 were not detailed. As stated above, the IPCC has not documented that agricultural intensification can regenerate degraded land [8,10,11,13]. For example, in the context of “Demand management” in “Climate Change and Land,” the IPCC referenced an article in *Science of The Total Environment* titled “Reducing the environmental impact of global diets” by Swain et. al., which stated in the abstract: "Modern, intensive livestock systems, especially for beef, offer substantially lower land requirements and greenhouse gas emissions per kilogram of meat than traditional, extensive ones. The land sparing potential of beef sector intensification is especially relevant for high-priority conservation regions like the Brazilian Amazon" [15]. In email correspondence, an IPCC agriculture author would not retract this reference extolling confined animal feeding operations to raise cattle in cleared Amazon Forest land from the IPCC report.

IPCC data on status quo global GHGs versus GHGs with regenerative/organic agriculture

Table 1 below [3] shows the status quo GHGs to be compared with GHGs with a global scenario of doubling global livestock and worldwide regenerative/organic agriculture.

**Table 1:** Net anthropogenic emissions (annual averages for 2010–2019) from AFOLU. AFOLU=Agriculture, Forestry and Other Land Use; GHG=Greenhouse gas; CO_2_=Carbon dioxide; CH_4_=Methane; N_2_O=Nitrous oxide; GTCO_2_ yr^–1^=Gigatonnes of carbon dioxide per year Positive values represent greenhouse (GHG) emissions into the atmosphere, negative values represent CO_2_ sequestration in the ground.

Anthropogenic						Natural Response	Natural + anthropogenic
Gas	Units	AFOLU Net anthropogenic emissions	Non-AFOLU anthro-pogenic GHG emissions	Total net anthro-pogenic emissions (AFOLU + non-AFOLU) by gas	AFOLU as a % of total net anthropogenic emissions, by gas	Natural land sinks including natural response of land to anthropogenic environmental change and climate variability	Net-land atmosphere CO_2_ flux (i.e. anthropogenic AFOLU + natural fluxes across entire land surface
		A	B	C=A+B	D=(A/C) x 100	E	F=A+E
CO_2_	GTCO_2_ yr^–1^	5.9 ± 4.1	36.2 ± 2.9	42.1 ± 29.0	14%	–12.5 ± 3.2	–6.6 ± 4.6
CH_4_	GTCO_2_e yr^–1^	4.2± 1.3	5.9 ± 1.8	10.2 ± 3.0	41%		
N_2_O	GTCO_2_e yr^–1^	1.8 ± 1.1	0.8 ± 0.5	2.6 ± 1.5	69%		
Total GHGs	GTCO_2_e yr^–1^	11.9 ± 4.4	44.0 ± 3.4	55.9 ± 6.1	21%		

In Table 1, the net land-atmosphere CO_2_ flux due to the natural response of land to climate and environmental change is shown for CO_2_ in column E. This is a nature-based buffer against global warming. Fossil fuel is the principal non-AFOLU anthropogenic CO_2_ source in Column B. Anthropogenic sources of methane (CH_4_) include rice agriculture, livestock, landfills and waste treatment, some biomass burning, and fossil fuel combustion. According to World Wildlife, “Rice, one of the most abundant crops grown and consumed globally, makes up 12% of global methane emissions” [16]. Rice produces about 1.2 GTCO_2_e /yr^-1 ^(0.12 * 10.2 GTCO_2_e/yr-1 (total net CH_4_) = 1.2 GTCO_2_e /yr-1). Rice is the largest source of CH_4_ after ruminant emissions. Consequently, no more than 3.0 GTCO_2_e /yr-1 of agriculture methane comes from livestock (4.2 GTCO_2_e /yr-1 - 1.2 GTCO_2_e /yr-1 (rice related) = 3.0 GTCO_2_e /yr-1). Natural methane emissions include CH_4_ emitted from sources such as wetlands, oceans, forests, fire, termites, and geological sources. Anthropogenic methane (CH_4_) is also emitted during the production and transport of coal, natural gas, and oil.

Global regenerative/organic agriculture’s potential for carbon sequestration in the ground

Paustian et al. reviewed published estimates of global soil carbon sequestration potential, representing the biophysical potential for managed cropland and/or grassland systems to store additional carbon assuming widespread (near complete) adoption of best management practices (BMPs). The majority of studies suggest that 4-5 GTCO_2_/yr is an upper limit for global biophysical potential with near complete adoption of BMPs [17].

If applied to the 5 billion hectares of mostly degraded agricultural land globally, 4 GTCO_2_-eq yr^-1 ^could regenerate enough land to sequester an additional 20 GTCO_2_-eq yr^-1 ^(4 t ha^-1^ yr^-1^ * 5 billion ha ≈ 20.0 GTCO_2_-eq yr^-1^). Table 2 below shows GHGs modeling with optimistic assumptions. Climate change would be at least paused even with more realistic assumptions.

**Table 2:** Net anthropogenic emissions with global AFOLU regeneration, including doubling livestock, optimizing forest management, and eliminating the burning of fossil fuels. AFOLU=Agriculture, forests, and other land uses; GHG=Greenhouse gas; CO_2_=Carbon dioxide; CH_4_=Methane; N_2_O=Nitrous oxide; GTCO_2_ yr^–1^= Gigatonnes of carbon dioxide per year

Anthropogenic						Natural Response	Natural + anthro-pogenic
GHG	Units	AFOLU Net anthro-pogenic emissions	Non-AFOLU anthropogenic GHG emissions	Total net anthropogenic emissions (AFOLU + non-AFOLU) by gas	AFOLU as a % of total anthro-pogenic emissions, by gas	Natural land sinks including natural response of land to anthropogenic environmental change and climate variability	Net-land atmosphere CO_2_ flux (i.e. anthropogenic AFOLU + natural fluxes across entire land surface
		A	B	C=A+B	D=(A/C) x 100	E	F=A+E
CO_2_	GTCO_2_ yr^–1^	-27.3	36.2 ± 2.9	8.9	NA	–12.5 ± 3.2	–39.8
CH_4_	GTCO_2_e yr^–1^	7.2	5.9 ± 1.8	13.1	NA		13.1
N_2_O	GTCO_2_e yr^–1^	1.8 ± 1.1	0.8 ± 0.5	2.6 ± 1.5	NA		2.6
Stop burning fossil fuels	GTCO_2_e yr^–1^		- 36.2	-36.2	NA		
Total GHGs	GTCO_2_e yr^–1^	- 18.3	6.7	- 11.6			-24.1

AFOLU net anthropogenic emissions (Column A, CO_2_) include regenerative/organic agriculture to sequester 20 GTCO_2_ ha^-1 ^yr^-1 ^(-4 GTCO_2_ ha^-1 ^yr^-1 ^[17] * 5 billion ha^ -1^ yr ^-1 ^= - 20.0 GTCO_2_ yr ^-1^). In addition, according to the IPCC [18], better forest, grassland, wetlands, and savannah management globally will sequester 7.3 GTCO_2_ yr^-1^ (3.9 GTCO_2_ yr^-1^ - 13.1 GTCO_2_ yr^-1^ range). This totals -27.3 GTCO_2_ yr^-1 ^ leaving the atmosphere and becoming sequestered in land (Column A at top). Finally, net-land atmosphere CO_2_ flux (i.e. anthropogenic AFOLU + natural fluxes across the entire land surface, (Column F for CO_2_) is the sum of Columns A and E (-39.8 GTCO_2_ yr^-1^ ≈ -27.3 GTCO_2_ ha^-1^ yr^-1 ^(Column A with CO_2_) - 12.5 GTCO_2_ ha^-1^ yr^-1^ (Column E with CO_2_).

As noted above in the explanation of Table 1, the portion of methane (CH_4_) from livestock is no more than 3.0 GTCO_2_-eq yr^-1^ since rice patties account for 1.2 GTCO2-eq yr^-1^. So in Table 2 Column B, the anthropomorphic CH_4_ from livestock doubled (3.0 GTCO_2_-eq yr^-1 ^* 2 = 6.0 GTCO_2_-eq yr^-1^) added to 1.2 GTCO_2_-eq yr-^1 ^(non-livestock agriculture CH_4_ from rice patties) =7.2 GTCO_2_-eq yr^-1^. The total CH_4_ in Column F (13.1 GTCO_2_-eq yr^-1^) comes from Column C as does the total NO_2_ (2.6 GTCO_2_-eq yr^-1^). The NO_2_ remained stable (NO_2_=2.6 GTCO_2-_eq yr^-1^ (Column C) compared with Table 1 because doubling the production of livestock allowed for the elimination of chemical fertilizers in switching to regenerative/organic agriculture.

Fossil fuels burning generated 36.2 GTCO_2_ yr^-1^ globally so when we stop burning fossil fuels (Column B), total anthropogenic GHGs ≈ -11.6 GTCO_2_-eq yr ^-1^ (Derivation: 8.9 GTCO_2_-eq-yr^-1 ^(Column C at the top) + 13.1 GTCO_2_-eq yr^-1 ^(Column C with CH_4_) + 2.8 GTCO_2_-eq yr^-1^ (Column C with NO_2_) - 36.2 GTCO_2_ yr^-1 ^(Column C with stop burning fossil fuels) ≈ -11.6 GTCO_2_-eq yr ^-1 ^). With the inclusion of net-land atmosphere CO_2_ flux (i.e. anthropogenic AFOLU + natural fluxes across the entire land surface) the net sequestration of CO_2_ totaled -24.1 GTCO_2_-eq yr ^-1 ^(Derivation: -39.8 GTCO_2_ yr-1 (Column F at the top) + 13.1 GTCO_2_-eq yr^-1 ^(Column F with CH_4_) + 2.8 GTCO_2_-eq yr^-1 ^(Column F with NO_2_) ≈ -24.1 GTCO_2_-eq yr ^-1 ^(Column F-total GHGs).

The net effect on global GHGs of regenerative/organic transitioning of agriculture, forests, and other land uses together with even reducing fossil fuel burning by half would be to pause if not significantly begin to reverse global warming. For each hectare of agricultural land regenerated with soil organic matter, it will take 20 to 30 years to reach saturation of sequestered CO_2_ [17]. The proposed modeling intervention of transitioning to global regenerative/organic agriculture (5 billion hectares) and doubling the global livestock for human consumption and land fertilization corresponds to net global GHGs = -24.1 GTCO_2_-eq yr^-1 ^only for 2-3 decades, totaling -482 to -723 GTCO_2_-eq, before reaching a new equilibrium with no further significant net carbon sequestration in the soil. If the transition to regenerative/organic agriculture globally begins to occur over 2-10 years, the maximum land CO_2_ saturation will be achieved over 40 or more years.

## Discussion

The need to double the global livestock population to reduce global early deaths from NCDs (<70 years old) is quite counterintuitive. I had been a vegan for 24 years when I saw my population-weighted and formatted worldwide Global Burden of Disease data on the inverse relationship between animal food consumption and NCDs. More context on this issue is needed.

George Monbiot, Guardian Opinion writer and author of “Regenesis: Feeding the World Without Devouring the Planet”[19], claimed that vegan humanity is the only possible salvation from climate catastrophe. Monbiot contended that organic agriculture and ranching damage the environment and foster global warming by being unproductive relative to conventional high-input, chemical agriculture. Prominent climate activists, including Greta Thunberg and Bill McKibben, endorsed Regenesis [19].

The IPCC report frequently referenced the negative climate consequences of high animal food consumption and never mentioned the negative health or climate consequences of low animal food intake. The IPCC report did not model increasing or decreasing global livestock [3]. Carbon footprint apps all consider a vegan diet the most climate-friendly option because of the methane emitted by ruminants. On the other hand, there is a strong nature-based case for the omnivore diet being the most healthful for humans. For instance, there has never been a report of a human who consumed an exclusively vegan diet from weaning from breast feeding until old age.

A case report by Dr. William Harris and myself published in Nutrition Journal [20] illustrates the danger of an exclusively vegan diet in infancy and early childhood. In 2006 while still a vegan, I provided pro bono medical research for an attorney representing a raw vegan couple in Florida who lost a 5½-month-old baby that weighed only seven pounds. After hearing that the parents fed the infant a raw vegan diet after only 2½ months of exclusive breast feeding, the district attorney charged the couple with manslaughter, incarcerated them, and sent their four older children into foster care. The four children had body mass indexes that ranged from 2-4 standard deviations below the mean for their ages, all qualifying for the World Health Organizations designation of severely underweight [21]. The children were otherwise healthy.

On studying the infant’s autopsy report, we concluded that the dead infant had a rare genetic malformation (DiGeorge Syndrome). The autopsy showed no thymus gland and fragmentary parathyroid glands, which are characteristic of this disorder. About 75% of DiGeorge Syndrome babies die within one year [22]. The judge found the couple innocent of manslaughter.

Despite the low weights of the older children, we successfully challenged the prosecution’s argument that the four older siblings were malnourished or otherwise abused. The judge reunited the family. The district attorney did not point out that the older children all had seriously low levels of vitamin B12, a vitamin essential for blood and neurological development. With negligible exceptions, vitamin B12 is available only in animal foods or in supplement pills. Fortunately for the parents and children, the court ordered that the remaining children had to eat an omnivore diet under the supervision of child protective services.

As described in the Journal of Nutrition [23] by evolutionary biologist Katharine Milton from the University of California, Berkeley, "Without routine access to animal-sourced food, it is highly unlikely that evolving humans could have achieved their unusually large and complex brain while simultaneously continuing their evolutionary trajectory as large, active and highly social primates. As human evolution progressed, young children, with their rapidly expanding large brain and high metabolic and nutritional demands relative to adults would have benefited from volumetrically concentrated, high-quality foods such as meat."

The IPCC numbers in Table 1 show that maintaining the GHGs status quo predicts a death sentence for humanity, especially since net anthropogenic GHGs have not yet begun to decline. Table 2 numbers represent a short-term, challenging but humanly possible scenario to pause global warming. Global land regeneration with soil organic matter would also dramatically enhance global food security and reduce early deaths of NCDs.

Global thought leaders, indigenous peoples leaders, nongovernmental organizations, philanthropists and climate activists, and the UN and national governments will need to figure out how to reduce global per capita GHGs. No high-technology-based intervention will be able to regenerate 5 billion hectares of agricultural land. Besides the approximately 2 billion farmworkers currently employed, it will take hundreds of millions more farmworkers and pastoralists. Regenerative/organic agriculture is more labor-intensive but less resource intensive than conventional agriculture. In developing countries, most poverty and malnutrition are among subsistence farming smallholder families (< 2 hectares/family). At the same time, smallholder families are more productive per hectare than larger farms that are more likely to use intensive technologies [24,25].

According to the United Nation’s website about the UN Sustainable Development Goals (SDGs), “Goal 13 calls for urgent action to combat climate change and its impacts. It is intrinsically linked to all 16 of the other Goals of the 2030 Agenda for Sustainable Development” [26]. Other SDGs include eliminating poverty, zero hunger, good health and well-being, and quality education for all. Attracting the additional needed farmworkers and pastoralists might possibly be accomplished with farmworker-owned cooperatives that offered additional livestock, assisted in transitioning to regenerative/organic agriculture, and committed to complying with all 17 of the UN’s SDGs. The huge process of doubling worldwide livestock and regenerating global land has the potential to be a win-win-win-win situation for the regeneration workers, the environment, the climate, and humanity. The requirements of transitioning to global regenerative/organic agriculture and meeting all the UN’s SDGs for farming families will require major financing and global human solidarity in accomplishing the tasks. However, when the global transition to regenerative/organic agriculture is completed 10-20 years after initiation, the farms and farmers will be financially sound because they will provide all the food to the world.

Mean carbon dioxide emissions per year now total about 7.4 t CO_2_ yr^-1^ per capita (59 GTCO_2_-eq yr ^-1^/ 8.0 billion people = 7.4 t CO_2_ yr^-1^) [3]. For global homeostasis, the anthropogenic GHGs should be no more than can be compensated for by the natural land carbon sinks including the natural response of land to anthropogenic environmental change and climate variability (i.e., 12.5 GTCO_2_ yr^ -1^, Table 2 Column E). After saturation of global land with sequestered CO_2_ in 40 or more years, reducing mean percapita GHGs to < 1.5 t CO_2_-eq yr^-1^ globally will be needed to keep atmospheric CO_2_ from increasing (12.5 GTCO_2_-eq yr ^-1 ^≈ 1.5 t CO_2_-eq yr^-1 ^* 8 billion people).

Limitations

The relatively minor anthropogenic GHGs (fluorinated gases (F-gases) comprising hydrofluorocarbons (HFCs), perfluorocarbons (PFCs), sulphur hexafluoride (SF6), as well as nitrogen trifluoride (NF3) [3] were not included in this analysis. A granular analysis of the potential of each of the 5 billion hectares of agricultural land regarding the potential for carbon sequestration was not attempted. Maximum regeneration potential including CO_2_ sequestration of forests beyond citing the modeling of the IPCC was not attempted. Likewise, the potential of returning oceans to sustainable conditions was not modeled. 

## Conclusions

The IPCC best climate case scenario (SSP1) would not sequester any net carbon in the ground, leaving the fate of humanity to experimental carbon-capturing technologies that have not yet been proven effective or practical. With the regenerative/organic agriculture scenario presented, retaining the existing farm workers and attracting hundreds of millions more workers would likely require transitioning most agricultural lands into worker-owned cooperatives that are committed to the UN's 17 Sustainable Development goals (e.g., eradicating poverty and zero hunger). Preventing human extinction could only be accomplished with the near elimination of the burning of fossil fuels and transitioning to global regenerative/organic agriculture in conjunction with accomplishing the UN's Sustainable Development Goals. The extent to which the current anthropogenic GHGs are out of balance with nature suggests that increasing the availability of renewable energy technologies needs to be together instead of lifestyle changes to radically decrease the overall human consumption of energy. After the saturation of global land with soil organic matter in 40 or more years after transitioning to regenerative/organic agriculture, the pause in global warming will end. Further human innovation will be required to adapt to a lifestyle with < 1.5 GTCO_2_-eq yr^-1^ per capita. This analysis presents a new scenario for preventing catastrophic global warming that should be analyzed by climate scientists. Their analyses of these findings, including the enumerated limitations, should be discussed with the media, indigenous people, policymakers, farmers, entrepreneurs, and the public.
